# Predictors of urethral stricture recurrence following urethroplasty: a retrospective review at the Jos University Teaching Hospital, Nigeria

**DOI:** 10.11604/pamj.2019.32.190.18504

**Published:** 2019-04-18

**Authors:** Chimaobi Gideon Ofoha, Venyir Mamhzi Ramyil, Nuhu Kutan Dakum, Samaila Ibrahim Shu'aibu, Idorenyin Cletus Akpayak, Felix Echebiri Magnus, Ushaaka Swem, Ayodele Oshagbemi, Olutayo Osunaiye, Julius Akhaine

**Affiliations:** 1 Jos University Teaching Hospital (JUTH), Nigeria; 2 College of Medicine, University of Jos, Jos University Teaching Hospital, Nigeria

**Keywords:** Urethra, stricture, recurrence, predictors, urethroplasty

## Abstract

**Introduction:**

Incidence of urethral stricture recurrence ranges between 2% to 36.4% with 75% occurring within the first 6 months of surgery. Hence, they need to identify the predictors of recurrence following urethroplasty.

**Methods:**

This is a retrospective study involving patients that had urethroplasty from January 2008 to December 2017. Patients' records were reviewed. Analyzed data were for patients with a minimum follow up of one year from the time of urethroplasty and included aetiology of urethral stricture, presence of suprapubic cystostomy, prior urethral dilatation, urine M/C/S, site of urethral stricture, length of urethral stricture, type of urethroplasty, level of training of the surgeon, type of urethral stent used and duration of stenting. Analysis was done using SPSS version 23. P-value of < 0.05 was considered significant.

**Results:**

Eighty seven urethroplasties were done, from January 2008 to December 2017. However, only records of 44 patients were accessible. Twenty patients completed duration of follow up ≥ one year. Urethral stricture recurrence was defined as resurgence of Lower Urinary Tract Symptoms (LUTS) within one year. Median age of the patients was 39.5 (± 19) years. Urethral stricture recurrence rate was 25% with mean time to recurrence from urethroplasty of 5.3 (±3) months. The use of preoperative suprapubic catheter (SPC) for urinary diversion as well as urethroplasties performed by the consultants had a lower incidence of recurrence.

**Conclusion:**

This study found urethral stricture recurrence of 25%. The level of training of surgeon vis-à-vis the expertise and experience seems to be an important factor, though not statistically significant in determining the outcome of urethroplasty.

## Introduction

Urethral stricture is one of the oldest diseases known to man. The term urethral stricture refers to anterior urethral disease and is a scarring process that involves the epithelium and the corpus spongiosum [1]. Contraction of the scar reduces the urethral lumen with subsequent development of lower urinary tract symptoms. In certain cases there is complete obliteration of the urethral lumen leading to urinary retention necessitating suprapubic cystostomy. Posterior urethral strictures are more correctly referred to as Pelvic Fracture Urethral Injuries (PFUIs). Strictures of the prostatic urethra or bladder neck are properly referred to as contractures or stenosis [1]. True world incidence of urethral stricture is difficult to come by in the literature. However an estimated incidence of 0.6% was reported in the United States of America [2]. In Sub-Saharan Africa, the incidence is probably higher, due to the higher prevalence of post infective urethritis in addition to other aetiologies. Treatment of urethral stricture has remained a challenge over the years with recurrent stricture being a major concern. Options for managing urethral stricture includes urethral dilation, internal urethrotomy and urethroplasty [3]. Amongst these, urethroplasty remains the gold standard today as it gives the greatest chance for cure in up to 91% [2]. Recurrence has been identified in several studies as one of the main complications following urethral stricture management, including urethroplasty. Incidence of urethral stricture recurrence ranges between 2% to 36.4% [2-5], with 75% occurring within the first six months of surgery [2]. There are varied definitions of urethral stricture recurrence in the literature. These include recurrent urinary symptoms and/or poor uroflowmetry <15ml/s, imaging showing the recurrence of the stricture [3], the requirements of additional intervention during follow up after the primary urethroplasty which may include dilatations, urethrotomies or redo-urethroplasty [3, 4] and failure to pass <16fr on cystoscopic assessment [2, 6]. Attempts have been made to identify some predictive factors for urethral stricture recurrence in the literature. Some of these factors include, preoperative, intraoperative and postoperative factors [7]. There are studies looking at the preoperative factors, however, there are few studies on intraoperative and post-operative factors that may predict stricture recurrence. Hence it becomes imperative to carry out a retrospective study looking at the conglomeration of factors that may predict urethral stricture recurrence as this may assist in guiding the management of urethral stricture as well as ensure an optimal outcome.

## Methods

This is a retrospective study involving patients that had urethroplasty (after adequate clinical evaluation) from January 2008 to December 2017. Patients' records were reviewed and data extracted. Analyzed data were for patients with a minimum duration of follow up of one year from the time of urethroplasty and included aetiology of urethral stricture, presence of suprapubic cystostomy, prior urethral dilatation, urine M/C/S, site of urethral stricture, length of urethral stricture, type of urethroplasty, level of training of the surgeon, type of urethral stent used (Silicon vs Latex catheter) and duration of stenting. Urethral stricture recurrence in this study was defined as resurgence of LUTS within one year of follow up from urethroplasty. Analysis was done using SPSS version 23. Measures of central tendencies; mean, median were done for the different variables. P-value of <0.05 was considered significant.

## Results

Eighty seven urethroplasties were done from January 2008 to December 2017. However, only records of 44 patients' were accessible, out of which 20 patients completed duration of follow up ≥ one year. Hence attrition rate was 55%. Prevalence of recurrence from this study was five out of 20 patients (25%). The mean time to recurrence from urethroplasty was 5.3 (±3) months. Age range was 14 years to 76 years with a median of 39.5 (±19) years. The effect of the study variables on recurrence of urethral stricture is shown in Table 1.

**Table 1:** Determinants of urethral stricture and urethral stricture recurrence in 20 patients

Determinants of urethral stricture	Frequency	Urethral stricture recurrence		P value
		Present %	Absent %	
**Aetiology of stricture**				
Trauma	13	4 (30.8)	9 (69.2)	0.417
Infection	7	1 (14.3)	6 (81.7)	
Total	20	5 (25)	15 (75)	
**SPC**				
No	1	1 (100)	0 (0.00)	0.076
Yes	19	4 (21.1)	15 (78.9)	
Total	20	5 (25)	15 (75)	
**Urethral dilatation**				
No	13	3 (23.1)	10 (76.9)	0.787
Yes	7	2(28.6)	5 (71.4)	
Total	20	5(25)	15 (75)	
**Urine MCS**				
Positive	13	3 (23.1)	10 (76.9)	0.787
Negative	7	2 (28.6)	5 (71.4)	
Total	20	5 (25)	15 (75)	
**Site of stricture**				
Bulbar	14	4 (28.6)	10 (71.4)	0.781
Bulbomembranous	5	1 (20)	4 (80.0)	
Penobulbar	1	0 (0.00)	1 (100)	
Total	20	5 (25)	15 (75)	
**Length of stricture**				
Short segment (≤2cm)	16	4 (25)	12 (75)	1.000
Long segment (>2cm)	4	1 (25)	3 (75)	
Total	20	5 (25)	15 (75)	
**Type of urethroplasty**				
Anastomotic	18	4 (22.2)	14 (77.8)	0.182
Substitution	1	0 (0.0)	1 (100.0)	
Staged	1	1 (100.0)	0 (0.0)	
Total	20	5 (25)	15 (75)	
**Level of training**				
Consultant	13	2 (15.4)	11 (84.6)	0.176
Senior Resident	7	3 (42.9)	4 (57.1)	
Total	20	5 (25)	15 (75)	
**Type of stent**				
Latex	7	1 (14.3)	6 (85.7)	0.417
Silicone	13	4 (30.8)	9(69.2)	
Total	20	5 (25)	15 (75)	
**Duration of stenting**				
≤2weeks	3	1 (33.3)	2 (66.7)	0.807
>2-≤4weeks	10	2 (20.0)	8 (80.0)	
>4-≤6weeks	2	1(50.0)	1 (50.0)	
>6weeks	5	1 (20.0)	4 (80.0)	
Total	20	5 (25)	15 (75)	

## Discussion

This study assessed the predictors of urethral stricture recurrence following urethroplasty in a sub-Saharan Africa setting. The proportion of stricture resulting from trauma is higher than strictures from post infective cause (Table 1). This is similar to reported urethral stricture aetiology in the literatures from sub-Saharan Africa [5, 8-10]. The rate of urethral stricture recurrence in this study was 25%, which is similar to that reported by Bello *et al.* [3] which puts the recurrence rate at 27.8%. A higher recurrence rate of 34.4% has been previously reported by Dakum *et al.* [5] working in the same centre in Jos, Nigeria. This lower recurrence rate observed between the index study and the previous one by Dakum *et al.* may have been due to refinements in techniques and acquisition of more experience. Though there is a trend towards lower recurrence rate in our centre, studies from other parts of the world show better recurrence rate. Andrich *et al.* [11] in his study had a recurrence rate of 12% at five years and 14% at fifteen years. This is similar to the finding by Meek *et al.* [4]. These discrepancies maybe explained by better instrumentation, refined techniques and expertise, thus further buttressing the place of expertise and refinements in techniques in determining the outcomes of urethroplasties for urethral stricture. The mean time to recurrence from urethroplasty was 5.3 (±3) months. This agrees with reported findings in the literatures where 75% of urethral stricture recurrence are seen within 6 months from urethroplasty [3, 8, 12, 13]. Identified possible causes of urethral stricture recurrence in this study (presence of suprapubic cystostomy, prior urethral dilatation, site of stricture, length of urethral stricture, type of urethroplasty, level of training, type of urethral stent and duration of stenting) were all not statistically significant (Table 1).

However the level of training of the surgeon seems to have an impact on the recurrence rate. Urethroplasty performed by the consultants had lower stricture recurrence compared to urethroplasty done by the residents, (15.4% vs 42.^9^%). This observation may be explained by the fact that the consultants have been in the urological practice longer than the residents and hence have more experience and technical skills. Bello [3] alluded to the place of expertise and volume in determining the differing recurrence rates in the literatures. Similarly Dakum *et al.* also highlighted the place of the surgeons expertise and ability in determining re-stricture rates [5]. It is pertinent to state at this point that, literatures directly evaluating the place of the surgeons' expertise, vis-à-vis the experience of the surgeons are sparse. Most studies focus mainly on preoperative and post-operative characteristics [2, 3, 6, 7, 14-17]. In this study, attempts have been made to identify some predictive factors for urethral stricture recurrence. However, limitations like the small sample size, the retrospective nature as well as the significant number of patients lost to follow up would have affected the outcome of the study. It is recommended that a prospective study with a large sample size will give a better picture of these predictors of urethral stricture recurrence after urethroplasty.

## Conclusion

This study found an overall urethral stricture recurrence of 25% with a mean time to recurrence from urethroplasty of 5.3 (±3) months. The analyzed determinants of recurrence were all not statistically significant. However, the use of preoperative SPC for urinary diversion as well as urethroplasties performed by consultants had a lower incidence of urethral stricture recurrence. Thus, the level of training of the surgeon vis-à-vis the expertise and experience seems to be an important factor, though not statistically significant, in determining the outcome of urethroplasty.

### What is known about this topic

- Urethral stricture is the narrowing of the urethra resulting in impedance to urine flow;
- Treatment options include dilatation, DVIU and urethroplasty;
- Recurrence rate is between 2%-40%.

### What this study adds

- Preoperative urinary diversion by suprapubic cystostomy and urethroplasties performed by consultants had lower incidence of recurrence of urethral stricture.

## Competing interests

The authors declare no competing interests.
